# Diseases of Louisiana

**Published:** 1833

**Authors:** 


ART. III. DISEASES OF LOUISIANA.

*The Application of Physiological Medicine to the Diseases of Louisiana.* By EDWARD H. BARTON, M. D. Honorary Member of the Philadelphia Medical Society, &c. &c. Philadelphia, 1832. pp. 55.

A friend has put into our hands a pamphlet bearing the above title. We place it at the head of our critical department, from respect for the design of the author. In doing this, we do not wish to be understood as pledging ourselves to the doctrines of the physiological school, but to the principle of manifesting respect for every one who has the enterprise to study the adaptation of new doctrines of any kind, to the diseases of the Great Valley; where so many physicians trudge on through a professional life of 30 years, without once stepping out of the beaten track of their predecessors.

The practice of our brethren of the lower Mississippi, is well known to be characterized by great boldness and energy. The lancet and calomel, particularly the last, constitute their anchor of hope, in the endemic fevers of that region. With this practice, Dr. Barton after ten or twelve years' experience, was by no means satisfied. He often saw it fail, and sometimes do harm; and, becoming a convert to the principles and practice of Broussais, resolved on testing their applicability to the fevers of the district in which he resided. The results of his trials are set forth in the little work now under review.

Not relying on the statement of these results, Dr. B. has gone into a somewhat elaborate course of pathological reasoning, to show, *a priori*, the adaption of the physiological practice to the diseases of which he treats. This does not appear to us the most efficient method which the case presented, and he would, we suppose, have been more successful as a propagandist, had he devoted a larger portion of his work to descriptions of the fevers of St. Francisville and its vicinity, and to the detail of practical facts and cases. Experience is the only test of medical opinion; and he who asks for the confidence of the profession in a new method, must show by diversified results, its advantages over those already in vogue.

As a specimen of our author's theoretical views, we shall extract the following paragraphs on the *modus operandi* of the remote causes of fever.

"It is acknowledged by physiologists, that the skin is the most extensive of the sensitive surfaces; and that in some animals it is, in fact, the only sense—that it is also the most important, must be equally acknowledged, from the universal care so assiduously employed by civilized nations in protecting it; from the many modes invented by the ingenuity, or discovered by the experience of man, in adding to its numerous enjoyments. Upon this important surface are developed parts of two systems of nerves, one part connecting it with the cerebro-spinal system, the other with the great system of organic life—the ganglionic or sympathetic system. Upon the former, which is merely the system of relation, by which man is connected with the external world, and though many of our enjoyments, as well as our pains are derived through it, yet noxious agents producing disease, but seldom, if ever, make their injurious influence on it. The latter system then being the great system of nutritive organic life, has an apparatus of nerves peculiar to itself, and that is appropriated to its peculiar functions—the *preservation of the individual;* of appropriating and converting into a part of ourselves, portions of the matter around us. It is made sufficiently clear by anatomical facts and demonstrations, as well as physiological and pathological deductions from them, that this apparatus pervades the whole system; it is sensitive to noxious as well as beneficial impressions, *without the immediate consciousness of the individual;* the centre of this system is the great abdominal brain; it is here that impressions made upon the periphery—its feelers—are repeated like that through the senses, upon the brain. If they are beneficial, the individual is only sensible of it in the improvement of his health, and that feeling of *well-being* inseparable from that state; if otherwise, we recognise it in the impairment of some function, whose co-existence is necessary for the continuance of health and the preservation of life. The broken harmony in the extensive chain of nutritive actions associated together in sympathetic relation, extends from system to system; where it is confined to the organic system, and the constitution is unable to resist its progress, and no means are used to assist it, the health of the individual is gradually undermined without exciting his apprehensions, feeling no pain, having no fever, the foe is not perceived; though by and bye, he feels that his energies are impaired, and the foundations of his constitution sapped. Here then is an impairment of the vital functions through the organic system alone, and as this is the system of support, as well as of increase and preservation, and as animal life depends upon it for its well-being and continuance, it likewise becomes affected secondarily. But noxious impressions are sometimes so violent and powerful, as to be extended to, and involve also the cerebro-spinal system, associated as they are together by an anastomosis with the pneumogastric, which is spread upon the gastric mucous membrane, and indirectly through the lateral ganglia. By the conversion of the physiological into the pathological state, pain is also experienced, which is not felt in their natural state; the sympathies are more and more awakened; the assaults of the foe are no longer concealed, and there is soon developed all the complicated phenomena of the diseased state.

"It is upon the gastric mucous membrane that this important system is chiefly developed, as this is the great and primary agent of nutritive, organic life; it is here is unfolded the great object and uses of that system; without it the individual could survive no longer. Instances are on record where every other organ has been wanting at birth, the stomach never. It is to the organic system what the head is to the intellectual: external impressions then are not only made upon the extremities of the organic system, but are repeated or felt immediately upon the extensive development of this system on the gastric mucus, and it is so on account of the especial uses and importance of this membrane. It partakes directly of all impressions on the system—it is influenced by the excitements of heat—it partakes of the tonicity of cold—a cold dampness debilitates both—a warm moisture relaxes both; a cathartic applied to the denuded skin purges—an emetic vomits—quinine strengthens—digitalis and strichnine make their peculiar impressions—emollient fomentations allay the irritability within, quench thirst and lessen excitement. It is generally admitted that the effect of applying medicinal agents to the skin arises from their agency upon the nerves of the part; these nerves are either in relation with the cerebro-spinal system or ganglionic. It is probable that vicissitudes of temperature act only upon the former—while these, and all agents that affect us, act upon the extremities of the latter, and thence their impression is conveyed to that organ or surface that has the most extensive, important and pervading sympathies—the gastric mucous,—unless their qualities are in special relation to the organic sensibility of some part, (and the more extensive these sympathies are, the greater the number it receives from other organs,) hence then it is implicated in all extensive irritations."

We shall make one or two additional excerpts, for the purpose of disclosing the pathological and therapeutic views, of which our author is the advocate, and which he regards as in keeping with the speculations which we have just presented.

"Believing then, that the great system of organic life, is the one which receives almost all impressions of disease, and particularly that most important part of it which is spread on the gastro-intestinal mucus, deriving its impressions through the direct and intimate associations with the epidermis; believing, that for the continuance of health, there must exist a due balance and equilibrium of power and harmony of action in the various parts of the system; that the primary impression of disease is local in the very nature of things, (to apply a cause to the whole system at once is incompatible with the physiological life of the individual, and too absurd for serious argument,) the part becomes irritated, a pathological state ensues, the organ most associated with it in function, in physiological importance, in periodical action at the time, or that may be in a state of sub-irritation, takes on the action or removes and translates it, and excites the sympathies, and effects ensue in proportion to the violence of the exciting cause, and number of the organs influenced by it. The theory of cure, then, may be resolved into one of antagonizing powers, a THEORY OF REVULSIONS, and that as all disease must consist of irregularity of action, a minor grade of action in one part is compensated by excess in another, the indication of equalizing is not so much accomplished by raising the first as by reducing the last; that mode is by depletion from whence the excess exists. DEPLETION then, is the primary agent in revulsion."

"The theory, then, is a local impression producing a pathological state, depending for its phenomena on an increase or exaggeration of local action. The constitutional irritation is the consequence of this, through association of action, and as however great constitutional excitement may be, it never can, in the nature of things, be every where equal. There must be grades of action, or of suffering; the medication must be to lessen the general amount by general bleeding, and as we cannot act upon a diseased organ directly—as to a highly irritated and excited organ, all internal remedies must at first be alike irritating, to a certain extent—the mode of relief is to lessen the amount of irritation in the organ most diseased, by local capillary depletion, or translate it to another organ in sympathizing connexion with it, and thus antagonize this organ against it, provided we can safely excite its depletory functional action by giving such means as act upon its organic sensibility. But when the sympathies are gone —the links that connect organ to organ, and in the aggregate form the *tout ensemble*, the organic life of the individual—there is no mode but the first. If the stomach is affected with a deeply radicated inflammation, this is not sufficient, and it may ameliorate, it may lessen the amount of its inflammation; but if the recuperative energies of the stomach are gone; if it has not the power of disembarrassing itself, by calling another organ sufficiently to its relief, death is inevitable. And even when the sympathies are not gone, they are often insufficient to remove a deeply radicated inflammation of the stomach. Such happens in our highest grades of algid fever, (and doubtless in cholera,) when, so far as my dissections have gone, the stomach is usually one coat of scarlet, and always extensively inflamed. Capillary depletion, then, is sufficient to lessen the action, (where it does not remove it altogether,) until it comes within the grade of a safe remedial power; we then call in a secretory action to its relief, by revulsing on it with some greater degree of permanence, and the system is thus enabled to re-act, and restore the lost balance and harmony."

On the subject of local blood letting, the *remedium summum* of the physiological school, he holds the following language:

"Were I to say then, that there is a *point* where local bleeding in fever is more particularly indicated than any other; that in fact it is as much the peculiar remedy, as when the respective remedies are called for the "blistering point," "bleeding point," &c. I would say that it is especially required when the abdomen is meteorized; *where there is tension of the epigastrium; a tension accompanied or not by pain;* there may be prostration of strength; there may be violent fever; there may be diarrhœa; there may be constipation; medicines rarely ever act here kindly or give relief. In corpulent subjects, it is of little comparative benefit, except from the epigastrium alone, from causes previously explained, and of the two modes, leeching with them, is much better than cupping."

Our author speaks in a sensible manner of cathartics, emetics and sudorifics. To the first, especially, he devotes several pages. He considers the *pros* and *cons*, but dwells longest on the latter, and if not an enemy to cathartics, is certainly not a friend. In our last number we delivered our own sentiments on this subject, and shall not here repeat them. We do not enter into all Dr. Barton's fears, but we feel disposed to recommend a perusal of what he has written to those physicians, who, in febrile diseases, make it a rule, daily, to *catharticise* their patients.

"It is not to be concluded from what has been said, that I am altogether unapprised of the great value of cathartic medicines. My objections are to their indiscriminate application; and I have freely stated the grounds of these objections, and trust they will be liberally examined by my brethren. As aperients to remove ingesta, colluvies from the bowels, and some cases of light irritation, as modes of acting upon the secretions, when that can be done with safety, as revulsive irritants and derivatives in a numerous class of important diseases, they form some of the most valuable weapons in the armory of the profession."

Of blisters our author entertains but a poor opinion. He thinks epigastric blistering in fever, generally injurious. We are "pretty much" of the same opinion.

His views in relation to sudorifics are not far out of the way.

"The best sudorific is to remove that state of internal irritation or inflammation to which the dryness and heat of skin are attributable, and local bleeding in the vicinity of the diseased organ, and cold applications, are the most immediate and direct."

He has devoted a number of pages to mercury, the liberal use of which he condemns. He thinks that medicine beneficial in proportion as it promotes secretion. On his brethren of the south, who sometimes give it in tea spoonful doses, he is even severe. He declares that such doses are not merely useless, but actually pernicious.

"There are two modes then for mercury to act upon the system, one upon the glands, the other upon the mucous membranes. When upon the first, it acts revulsively, the depletion, (secretion,) often relieves the suffering, the irritation or inflammation for which it was given, and benefits result from its use. When, however, no gland sympathises or relieves the organ receiving the impression, it acts as a poison upon the part.

But does salivation in fever always produce safety? Is it even an evidence that all danger is over? We know it is not in other diseases. Experience has fully solved this question. Numerous are the cases on record, and many more are known to practical men, of its being no indication whatever, of ptyalism appearing in the intermission or remission, and subsiding with the occurrence of the paroxysm, evincing its little power, and proving the intermittent nature of inflammation, whether we take that of the disease or the remedy; of patients dying salivated; of many often attacked with bilious, yellow, and other fevers, while salivated for the venereal, or for the express view of warding off an attack; of a free and full ptyalism having no influence whatever on yellow fever, of which I have seen many instances. In fact, it is no better than any other revulsion, whether a purge, blister, or sinapism; nor so good, for with two of these we can better control the time, place, and circumstances. It produces no greater assurance of safety, while a thousand objections will apply to its indiscriminate administration; in the various and complicated affections incident to its use; never certain in its effects, but always liable to be abused, while most others can be used, if not always certain in their influence, there is little jeopardy of their after effects."

We have read Dr. Barton's little work with pleasure and profit. As a writer he is inexperienced, but we hope to hear from him again.

